# Editorial: Explaining and comparing ethnic and racial discrimination

**DOI:** 10.3389/fsoc.2024.1417315

**Published:** 2024-08-09

**Authors:** Pieter-Paul Verhaeghe, Mariña Fernández-Reino, Valentina Di Stasio

**Affiliations:** ^1^Brussels Institute for Social and Population Studies (BRISPO), Vrije Universiteit Brussel, Brussels, Belgium; ^2^Centre on Migration, Policy and Society, University of Oxford, Oxford, United Kingdom; ^3^European Research Centre on Migration and Ethnic Relations (ERCOMER), Utrecht University, Utrecht, Netherlands

**Keywords:** racism, discrimination, correspondence tests, attitudes, rental discrimination, hiring, education, policy

Research on the causes and consequences of ethnic and racial discrimination is central to social, economic, and psychological sciences. Discrimination is defined as the unequal and unfair treatment of people because of their (perceived) ethnic or racial origin. While US scholars have focused on race-based discrimination, European scholars have paid more attention to discrimination on grounds of ethnicity, nationality and religion. Historically, race has structured intergroup relations in the US case due to the legacy of slavery and (neo-)colonialism. In the European context, on the other hand, religion and Islam have increasingly been identified as the main boundary of exclusion and racialization (Alba, 2005; Bail, 2008). In Europe too, though, recent scholarship has called for a stronger focus on the formation and maintenance of racial hierarchies, also independent of religion (Osanami Törngren and Suyemoto, 2022; Polavieja et al., 2023).

Research on levels and patterns of discrimination in rental markets (Flage, 2018; Auspurg et al., 2019) and hiring (Zschirnt and Ruedin, 2016; Quillian and Midtbøen, 2021; Lippens et al., 2023) is often based on field experimental techniques such as correspondence and audit tests, widely considered as the gold standard to measure discriminatory behavior (Heath and Di Stasio, 2019; Verhaeghe, 2022). Another research tradition makes use of lab and survey experiments, such as vignettes, behavioral games or implicit association tests, to examine the drivers and moderators of individuals' biased behavior (Lane, 2016). A last research stream, based on surveys or interviews, focuses on perceived discrimination by ethnic minority groups (Schaeffer and Kas, 2023).

Notwithstanding these rich and growing traditions, there is still little interdisciplinary dialogue between them. This is unfortunate, as it is only through the combination of methodologies and theoretical perspectives and the comparison of settings and countries, that we could get a richer and deeper understanding of how, where and why discrimination occurs, and its impact on individuals and society.

In this Research Topic, we promoted a dialogue between the different research streams on discrimination. The seven articles rely on a variety of methodologies, and cover different settings (housing, schools, labor, health care, the police and courts) and regions (Belgium, Germany, Hungary, Sweden, the U.S., and Europe as a whole). Unfortunately, several of these studies found evidence of persistent discrimination in rental applications, hiring, the access to education, and encounters with the police. Based on correspondence audits in the rental housing market, Váradi et al. revealed widespread and socially acceptable practices of discrimination against Roma people in Hungary, while Martiniello and Verhaeghe found significant discrimination against applicants with Moroccan- and Polish-sounding names in Belgium. With respect to hiring preferences, Osanami Törngren et al. demonstrated that recruiters in Sweden rate candidates with Chinese names more favorably than those with Iraqi names. In Germany, Kogan et al. showed that male adolescents from the Middle East and Africa report more police discrimination than their female peers and other ethnic minorities. These discriminatory experiences result in lower levels of trust in the police and courts. Finally, Ramos Lobato et al. found that there are several dimensions of institutional discrimination in the access to schools in Germany, resulting in exacerbated social and ethnic school segregation.

On a more positive note, some studies in this Research Topic did not find unequal treatment or prejudicial attitudes toward ethno-racial groups in specific situations. In their conjoint survey experiment on racial biases against doctors in the United States, Olinger et al. did not find any significant prejudice against doctors from minoritized ethnic groups, a result that holds irrespective of the racial background of patients. In addition, Osanami Törngren et al. used eye-tracking and dialogic data to investigate the way recruiters from diversity-friendly organizations rate CVs. Probably due to this specific sample, non-White CVs were rated more favorably than White CVs. In general, we are happy to provide an outlet for above non-significant differences too. It is only by also analysing these cases with no or much less discrimination, that we could get a deeper understanding of overall patterns of discrimination.

The articles collected in this Research Topic exemplify the methodological versatility of discrimination research: the methods they rely on range from interviews (Ramos Lobato et al.) and focus groups (Váradi et al.) to conjoint experiments (Olinger et al.) and quantitative analysis of survey data (Blommaert and Coenders; Kogan et al.), with some combining the experimental rigor of experimental designs with qualitative analysis of dialogs (Osanami Törngren et al.) and survey data (Martiniello and Verhaeghe). Importantly, using this combination of methods is promising, as it could shed light on the underlying mechanisms of discrimination. These mechanisms could differ across settings. A common aspect highlighted in a few of the articles is the influence of norms, perceptions and practices beyond individual behavior, which is often driven by group stereotypes and sustained by institutional practices that are often taken for granted in spite of the possibly unequal outcomes they lead to. In their study on rental discrimination, Martiniello and Verhaeghe, for example, combine correspondence tests with a survey about the perceptions of names. They found that real estate agents especially discriminate against minority candidates when their names are perceived as religious (conceptualized by the authors as “religious taste-based discrimination”), whereas landlords discriminate more against candidates with non-European names (labeled as “ethnic taste-based discrimination”). Furthermore, Ramos Lobato et al. show through a combination of surveys and expert interviews that institutional discrimination against ethnic minority students can be explained by the interplay of parental school choices and the institutional structures of the school. The study highlights how vaguely defined admission criteria and unclear implementation guidelines provide school principals and admission officers with substantial leeway, resulting in preferential treatment of middle-class white students.

Finally, we are glad that we could present a few studies that elaborate on potential interventions to tackle discrimination. Blommaert and Coenders, for example, examine public support for three types of diversity policies based on a representative survey of the European population. They found relatively stronger support for diversity training and monitoring recruitment procedures, compared to more preferential and prescriptive policies, such as monitoring the workforce composition. Importantly, national legislation can contribute to raising awareness and changing social norms, as public support for all three types of policies examined was larger in countries with more progressive anti-discrimination laws. Finally, the qualitative study of Váradi et al. suggests that prejudices among Hungarian landlords about Roma tenants might be challenged on an emotional level by providing counter-information. These findings are hopeful because they offer policy makers concrete avenues to combat discrimination.

This Research Topic makes clear that discrimination is not a setting-specific experience but a structural phenomenon that cannot be merely reduced to the biases of single decision-makers (Blank, 2005; Reskin, 2012; Small and Pager, 2020). We see much promise in future work that directly compares levels and patterns of discrimination across settings, that examines the cumulative impact of discrimination during the life course and over generations, and that can quantify how direct and indirect or institutional discrimination relate to, and magnify, each other.

## Author contributions

P-PV: Conceptualization, Writing – original draft, Writing – review & editing. MF-R: Conceptualization, Writing – original draft, Writing – review & editing. VD: Conceptualization, Writing – original draft, Writing – review & editing.
